# Maternal Diabetes in Pregnancy: Early and Long-Term Outcomes on the Offspring and the Concept of “Metabolic Memory”

**DOI:** 10.1155/2011/218598

**Published:** 2011-11-21

**Authors:** Akadiri Yessoufou, Kabirou Moutairou

**Affiliations:** ^1^Centre for Integrative Genomics, University of Lausanne, Genopode Building, 5è Etage, 1015 Lausanne, Switzerland; ^2^Laboratory of Cell Biology and Physiology, Department of Biochemistry and Cellular Biology, Faculty of Sciences and Techniques and Institute of Biomedical and Applied Sciences (ISBA), University of Abomey-Calavi, 01 BP 918 Cotonou, Benin

## Abstract

The adverse outcomes on the offspring from maternal diabetes in pregnancy are substantially documented. In this paper, we report main knowledge on impacts of maternal diabetes on early and long-term health of the offspring, with specific comments on maternal obesity. The main adverse outcome on progenies from pregnancy complicated with maternal diabetes appears to be macrosomia, as it is commonly known that intrauterine exposure to hyperglycemia increases the risk and programs the offspring to develop diabetes and/or obesity at adulthood. This “fetal programming”, due to intrauterine diabetic milieu, is termed as “*metabolic memory*”. In gestational diabetes as well as in macrosomia, the complications include metabolic abnormalities, degraded antioxidant status, disrupted immune system and potential metabolic syndrome in adult offspring. Furthermore, there is evidence that maternal obesity may also increase the risk of obesity and diabetes in offspring. However, women with GDM possibly exhibit greater macrosomia than obese women. Obesity and diabetes in pregnancy have independent and additive effects on obstetric complications, and both require proper management. Management of gestational diabetes mellitus and maternal obesity is essential for maternal and offspring's good health. Increasing physical activity, preventing gestational weight gain, and having some qualitative nutritional habits may be beneficial during both the pregnancy and offspring's future life.

## 1. Introduction

Compelling evidence exists suggesting that exposure to an adverse fetal and/or early postnatal environment may enhance susceptibility to a number of chronic diseases in the future life of offspring. Gestational diabetes mellitus (GDM) and obesity are both complications which occur during pregnancy and substantially influence the development of offspring during fetal life and postnatally. Indeed, fetuses from mothers with gestational diabetes are at high risk of developing fetal macrosomia [1, 2]. Although most of the women with GDM return to normal glucose tolerance after delivery, they have an increased risk of developing diabetes, mainly type 2 diabetes mellitus [3]. Offspring of women with gestational diabetes are prone to adverse side effects such as macrosomia, which is strongly associated with fetal death, prematurity, birth trauma, and respiratory distress syndrome [4]. These offspring have a high risk of developing obesity, impaired glucose tolerance, and type 2 diabetes in adulthood [4]. The concern of most researchers, during the last decade, is to explore the physiopathology of the relationship between the health conditions of offspring born from pregnancy complicated with diabetes. Our team has evidence in many experimental studies, in which we have observed a high incidence of macrosomia in the litters of diabetic animals [1]. The macrosomic (large-sized) offspring of diabetic animals exhibit many physiological disorders associated with metabolic syndrome. However, the mechanisms by which excess maternal weight and/or diabetes during pregnancy may lead to disease in the offspring at childhood and adulthood are not fully understood. The aim of this paper is to summarize new knowledge on the various physiological and pathophysiological aspects of early and long-term offspring outcomes of maternal diabetes during pregnancy. Specific comments on impacts of maternal obesity on offspring health are also evoked, since the impact of obesity and GDM on fetus and mother often becomes circular, as the majority of mothers with GDM are obese and a significant proportion of those who are obese have GDM [5].

## 2. Gestational Diabetes: Meaning and Diagnosis

Depending on the diagnostic and screening criteria, it has been observed that prevalence of GDM ranged from 1.3% to 19.9% [6]. In obesity context, a meta-analysis [7] showed that the risk of developing GDM was 2.14-fold higher in overweight pregnant women, 3.56-fold higher in obese pregnant women, and 8.56-fold higher in severely obese pregnant women compared to pregnant women with normal weight. This analysis prompted the International Association of Diabetes and Pregnancy Study Groups (IADPSG) to propose new criteria for the diagnosis of GDM, based on the Hyperglycemia and Adverse Pregnancy Outcomes (HAPO) Study [8]. The criteria use a 75-g oral glucose tolerance test (OGTT) without prior glucose challenge and diagnose GDM when the fasting glucose is ≥5.1 mmol/L and/or when the 1-h postload glucose is ≥10.0 mmol/L and/or when the 2-h postload glucose is ≥8.5 mmol/L. 

With the greater number of pregnancies complicated with diabetes, it will be interesting to monitor the long-term impacts of maternal diabetes in pregnancy on the health condition of offspring.

## 3. Macrosomia: The Main Adverse Outcome of Diabetes in Pregnancy

### 3.1. Studies in Humans

Maternal diabetes is characterized by an increased placental transport of glucose and other nutrients from the mother to the fetus, resulting in macrosomia [9]. Convincing studies have shown that either preexisting diabetes (type 1 and type 2 diabetes) or GDM (diabetes only during pregnancy) are associated with macrosomia [10–17]. Indeed, epidemiological and clinical studies have shown that maternal type 1 diabetes during pregnancy is an important risk factor for fetal overnutrition and macrosomia and for the development of obesity and diabetes in offspring [10, 11]. Type 2 diabetes and GDM are also associated with macrosomia and diabetes in the progenies [12, 13]. The risk of diabetes in offspring of type 2 diabetes genitors is significantly higher when the mother rather than the father is diabetic [12]. Moreover, the risk of insulin resistance is higher in children of mothers with GDM (diabetes only during pregnancy) than in children from mothers developing diabetes after pregnancy [14]. Macrosomia, the most commonly reported effect of maternal diabetes in newborns [15], is usually defined in humans as birth weight above either 4 kg or birth weight above the 95th percentile of the gestational age. In human studies, 43% of GDM patients had a macrosomia history [16, 17]. In total, 75% of the diabetic mothers had an episiotomy during delivery. Babies from GDM patients whose birth weight was 2.0 SD greater than the mean birth weight of control infants were considered as macrosomic babies.

### 3.2. Animal Models

In animal studies, the model reported here concerns streptozotocin-induced type 1 diabetic pregnancy which also leads to macrosomia in offspring [18, 19]. Several modes exist for inducing diabetes with streptozotocin. The group of Van Assche has exhaustively investigated the consequences of experimental maternal diabetes induced by streptozotocin on fetus and adult progeny [9, 20]. 

The streptozotocin, when administered at a high single dose, induces diabetes by the direct toxic effects on pancreatic *β*-islet cells [9]. The fetus is confronted with severe intrauterine hyperglycemia which induces fetal islet hypertrophy and *β*-cell hyperactivity and may result in early hyperinsulinemia [20]. This overstimulation of fetal *β* cells limits their adaptation, and they become depleted of insulin granules [20], and incapable to secrete insulin [9]. *β*-cell exhaustion results in fetal hypoinsulinemia. Hypoinsulinemia and a reduced number of insulin receptors on target cells lead to a reduction in fetal glucose uptake [9]. The growth of fetal protein mass is suppressed, and fetal protein synthesis is consistently low, leading to fetal microsomia [9]. Postnatal development is retarded, and these offspring remain small at adulthood; however, they develop insulin resistance [9, 21].

 However, streptozotocin, administered at low doses during 5 consecutive days, induces mild type 1 diabetes, following a T-lymphocyte-dependent process, an autoimmune destruction of pancreatic *β* cells, mediated by both CD4^+^ and CD8^+^ T cells [22, 23]. The administration of low doses of streptozotocin to rodents represents a good model of diabetes development for several reasons [22, 24, 25]. The intrauterine mild hyperglycemia also induces fetal hyperinsulinemia with hypertrophy of the endocrine pancreas and hyperplasia of the *β* cells [20]. Animals with perinatal hyperinsulinemia display an impaired glucose tolerance at adulthood only under high glucose [9]. 

A model of diabetic pregnancy and macrosomia through administration to pregnant *Wistar *rats of five low doses of streptozotocin starting on day 5 of gestation is also well established [1, 26, 27]. Pups from diabetic pregnant rats whose birth weights were 1.7 SD greater than the mean birth weight of the control pups were considered as macrosomic offspring [1, 26, 27]. As far as the model is concerned, it is important to note that maternal streptozotocin administration before pregnancy affects fertility and impairs embryo development during preimplantation period [28]. However, the induction of diabetes by streptozotocin injection on day 5 of gestation [1] has no effect on embryo development [19]. We observed that 62% to 75% of pups born to diabetic pregnant rats were macrosomic at birth [1, 27, 29]. These macrosomic (large-sized) offspring of diabetic dams were hyperglycemic at birth and maintained an accelerated weight gain until the monitoring time of 12 weeks [26, 30], compared to offspring of control rats. 

Furthermore, maternal hyperlipidemia during diabetic pregnancy [1] has been shown to be one of the predisposing factors of macrosomia in offspring. In fact, high levels of triglyceride in maternal circulation of diabetic rats may create a steep concentration gradient across placenta, which accelerates their transport and deposition in fetal tissues [31]. In macrosomic offspring, this hypertriglyceridemia persists with age and is linked to the development of insulin resistance and hyperlipogenesis [32]. Besides, maternal hyperglycemia also leads to fetal hyperglycemia, which stimulates pancreatic islet cells and induces fetal hyperinsulinemia [26, 27, 32]. The intrauterine hyperinsulinemic state results in an increase of fat synthesis and body size [33]. The increase in body weight is a consequence of an increase in adipose tissue weight and lipid content at all ages. 

Thus, macrosomia appears as the main outcome of maternal diabetes, and both pathologies are associated with several metabolic disorders, implicating lipid metabolism and antioxidant status.

## 4. Main Metabolic Consequences during Maternal Diabetes and Macrosomia

### 4.1. Lipid Metabolism Is Altered during Maternal Diabetes and Macrosomia

#### 4.1.1. Animal Models

As far as lipid metabolism is concerned, experimental diabetes has been shown to impair maternal and fetal lipid metabolism [31, 34]. In experimental models, type 1 diabetic pregnancy in rats is associated with a significant increase in serum and hepatic triglyceride (TG) and total cholesterol (TC) [1, 27, 29]. Macrosomic and obese offspring of diabetic rats exhibit high adipose tissue weight, together with high adipose tissue lipid contents [27], and they show high serum and liver lipid levels [1, 26, 29, 30]. The hypertriglyceridemia and hypercholesterolemia, common features of experimental obesity, are the direct consequences of hyperinsulinemia and hepatic hyperlipogenesis [35, 36]. The major findings on fatty acid composition in adult macrosomic offspring were parallel with those of their diabetic mothers. Diabetic pregnancy causes a profound decline in plasma arachidonic acid (AA, C_20_ : 4n-6) and an increase in linoleic acid (LA, C_18_ : 2n-6) concentrations in rats and in their macrosomic and obese offspring [1, 29], and this may be due to an impaired activity of Δ5- and Δ6-desaturases enzyme [37]. Diabetes-induced low concentration of plasma AA may have a critical role in maintaining the appropriate mass and function of islet *β* cells by influencing rates of cell proliferation and insulin secretion [38, 39].

#### 4.1.2. Studies in Humans

Human studies revealed in GDM patients that diabetes appeared at second or third trimester of pregnancy [16, 17] as determined by oral glucose tolerance test according to the World Health Organization criteria. GDM patients were hyperglycemic and hyperinsulinemic at the diagnosis of the disease [16, 17], reflecting a decrease in insulin sensitivity in diabetic pregnant women [40]. Several studies including ours have shown that, when compared with normal values, GDM mothers as well as control mothers exhibited hypertriglyceridemia and hypercholesterolemia, throughout pregnancy, and no significant difference exists between healthy and diabetic women [16, 17, 40–42]. However, macrosomic babies showed high levels of serum triglyceride and total and free cholesterol compared with control infants [16, 17].

Thus, maternal diabetes and macrosomia induce an alteration in lipid metabolism.

### 4.2. Antioxidant Status Is Affected during Maternal Diabetes and Macrosomia

One of the earliest abnormalities observed in diabetic subjects is the involvement of oxidative stress [43]. Moreover, fetuses from mothers with gestational diabetes are at increased risk of developing platelet hyperaggregability and oxidative stress [2]. High blood glucose levels in these newborns induce oxidative stress [2], which, in turn, induces the production of highly reactive oxygen radicals, being toxic to cells, particularly to the plasma membranes where these radicals interact with the lipid bilayer. Endogenous antioxidant enzymes (e.g., superoxide dismutase, catalase, glutathione peroxidase, and reductase) and vitamins are responsible for the detoxification of deleterious oxygen radicals [44]. In diabetes as well as in macrosomia, protein glycation and glucose auto-oxidation may generate free radicals, which, in turn, catalyze lipid peroxidation [45]. Moreover, disturbances in the antioxidant defense system in diabetes and macrosomia have been reported as follows: alteration in antioxidant enzymes activities [46], impaired glutathione metabolism [47], and decreased ascorbic acid levels [48].

#### 4.2.1. Studies in Humans

In human studies [17], we assess the serum antioxidant status through antiradical resistance (KRL; Kirial International SA, Couternon, France) and levels of vitamin A, C, and E and activity of superoxide dismutase (SOD). GDM as well as macrosomia induce an altered total serum antioxidant defense status [17]. Indeed, gestational diabetic women exhibit decreased levels of vitamin E and enhanced concentrations of vitamin C without any changes in vitamin A. Macrosomia also induces decreased levels of vitamin E. GDM and macrosomia are also associated with impaired SOD activities and enhanced levels of serum thiobarbituric acid-reactive substances (TBARSs), suggesting an increased oxidative stress [17].

#### 4.2.2. Animal Models

In experimental model [1], type 1 diabetic pregnancy and macrosomia lead to a significant decrease in the plasma total antioxidant status as measured by diminished plasma oxygen radical absorbance capacity (ORAC) in diabetic pregnant rats and their macrosomic pups [1]. We have also observed increased plasma TBARS, decreased erythrocyte superoxide dismutase and glutathione peroxidase activities in diabetic rats and their macrosomic offspring, and diminished vitamin A levels in diabetic dams and vitamin C concentrations in macrosomic pups. Several authors have also shown diminished antioxidant enzyme activities and vitamin levels in streptozotocin-induced diabetic rats [46–48]. 

To sum up, in animals as well as in humans, maternal diabetes and macrosomia are associated with altered antioxidant status [1, 17].

## 5. Is Neonatal Obesity Programmed during *In Utero* Life? New Concept of a “*Metabolic Memory*”

The hypothesis on fetal origin suggests that the fetal malnutrition, which, during pregnancy, induces disruption in fetal growth and thinness at birth, programs latter type 2 diabetes and metabolic syndrome [49]. At critical and delicate period of fetal development, the process by which a stimulus induces long-term impacts on fetus, previously described and established as “*fetal programming*” by Hales and Barker [49], is termed as new concept of “*metabolic memory*.” In the same line, all the observed metabolic abnormalities among gestational diabetic women create an *in-utero* environment around the fetus which programs him to diseases during his adulthood [49, 50]. This *in utero programming* seems to create a kind of “*metabolic memory*,” since physiological anomalies of gestational period are responsible for the onset of diseases in offspring at adulthood, such as type 2 diabetes and obesity associated with metabolic syndrome. It is noteworthy that several alterations in carbohydrate and lipid metabolism, observed in infants of diabetic mothers at birth, also persist postnatally. As an example of this phenomenon of metabolic memory, we can mention a study of Palinski and Napoli [51] who demonstrated that maternal hypercholesterolemia during pregnancy is associated with greatly increased fatty streak formation in human fetal arteries and accelerated progression of atherosclerosis during childhood [51]. A good correlation exists between maternal and fetal plasma cholesterol levels in 5-6-month-old human fetuses [52, 53]. Moreover, maternal hyperglycemia has been shown to lead to fetal hyperglycemia which stimulates fetal pancreatic islet cells to produce fetal hyperinsulinemia [54]. The ability of fetal hyperinsulinemia to increase the availability of farnesylated p21-Ras may represent one of mechanisms of the growth-promoting action of insulin during fetal development [55]. 

**Figure 1:**
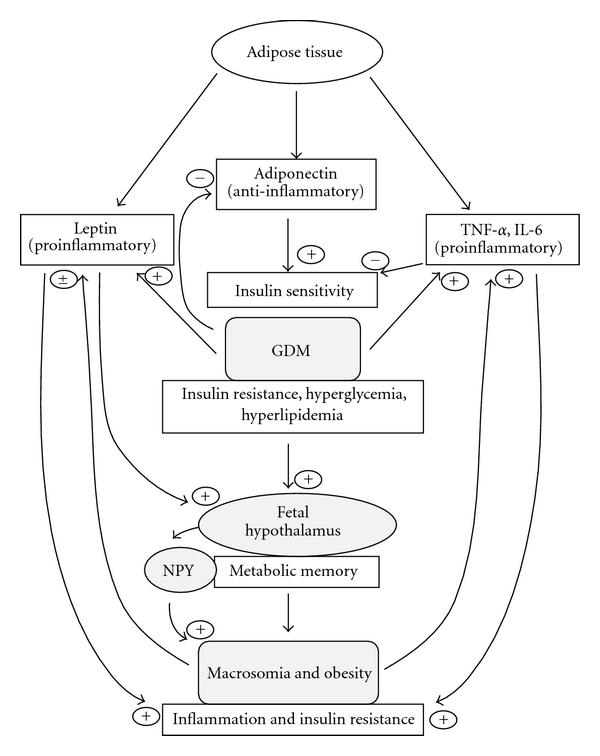
In GDM, adipose tissue secretes low adiponectin (anti-inflammatory and positive stimulator of insulin sensitizing) and high TNF-*α* and IL-6 which contribute to inflammatory state and insulin resistance in diabetic pregnancy as well as in macrosomia. Leptin, being pro-inflammatory, is highly produced by adipose tissue during diabetic pregnancy and insulin resistance (experimental study [75]) and implicated in the pathogenesis of weight gain in macrosomic babies. Leptin may exert its effects by interacting with neuropeptide-Y in the hypothalamus. The intrauterine hyperglycemia may act on the fetal hypothalamus and create a kind of “*metabolic memory*” which programs obesity and metabolic syndrome in the offspring during adulthood. (+) positive regulation (−) negative regulation. NPY, neuropeptide-Y.

Another example of *metabolic memory* is revealed by Franke et al. [56] who have shown that diabetic pregnancy in rats alters the differentiation of hypothalamic neurons of newborns (Figure 1). The alterations of hypothalamic neurons may be avoided by normalizing the glycemia among diabetic pregnant rats [56]. The increased levels of neuropeptide-Y (Figure 1) in offspring of hyperglycemic rats may be explained by a defected *programming* of the hypothalamic neurons, due to intrauterine environment of gestational diabetic milieu [56]. These alterations may increase the risk of trend in high food taking, overweight, obesity, and diabetogenic status in offspring at adulthood (Figure 1). All these observations prove an *in utero programming* of metabolic syndrome in offspring born to maternal diabetes.

## 6. Modulation of Insulin Resistance and Inflammation during Maternal Diabetes and Macrosomia

Gestational diabetes and obesity are two pathologies associated with insulin resistance and inflammation which are profoundly modulated by adipokines and cytokines [16]. Obesity is associated with high adiposity and hyperlipidemia [57]. Moreover, low-grade inflammation has been reported to be a link between insulin resistance, obesity, and type 2 diabetes [57]. Thus, it appears that inflammation may modulate insulin resistance in GDM.

### 6.1. Studies in Humans

There is evidence that hypoadiponectinemia is associated with pathogenesis of GDM and macrosomia [58]. Adipokines and cytokines, through their ability to interfere with insulin signaling, have been implicated in insulin resistance [59]. Adiponectin, a physiologically active polypeptide hormone derived from adipose tissue, exhibits insulin-sensitizing, antiatherogenic, and anti-inflammatory properties [60]. 

In human studies, we and other investigators have shown that women with GDM, compared with non-diabetic women, exhibited a decreased concentration of adiponectin (anti-inflammatory agent) [16, 61], concomitant with an increased concentration of TNF-*α* and IL-6 (pro-inflammatory cytokines) [16]. Is there any physiological crosstalk between the high levels of TNF-*α* and the low adiponectin concentrations in women with GDM? It has been shown that adiponectin and TNF-*α* produce opposite effects on insulin signaling, with inhibiting action of TNF-*α* [62] and increasing action of adiponectin [63] on tyrosine phosphorylation of the insulin receptor. Besides, it is also possible that TNF-*α* may be responsible for lowered synthesis of adiponectin in GDM subjects, as suggested by Lihn et al. [64] that TNF-*α* and IL-6 downregulate adiponectin expression (Figure 1). Regarding the long-term effect on the offspring of gestational diabetic women, it is important to mention the study of Tsai et al. [65], who have demonstrated that decreased maternal adiponectin concentration and insulin sensitivity may increase the risk of fetal overgrowth in women suffering from GDM. However, our study revealed that concentrations of TNF-*α*, IL-6, adiponectin, and leptin are decreased in macrosomic babies compared to control infants [16]. Furthermore, IL-6 has been shown to be one of the mediators of hyperinsulinemic state [66], 10%−35% of the body's basal circulating IL-6 is derived from adipose tissue, and a positive correlation has been found between insulin resistance and circulating IL-6 [57].

Leptin is not only produced by the placenta but principally by the adipocytes, secreted into the bloodstream [67], and involved in weight gain regulation and lipid metabolism. Leptin is an appetite-suppressant agent, and it exerts its effects by interacting with neuropeptide-Y in the hypothalamus (Figure 1) [68]. Contradictory results have been reported about leptin secretion during GDM and macrosomia. GDM is either associated with high levels of leptin [69], no change [70], or reduced level of leptin [71]. Our previous reports have shown high leptin level in mothers with GDM and reduced level of leptin in their macrosomic infants [16]. This discrepancy could be a result of the difference in the time of maternal blood collection (i.e., gestational age). However, elevated leptin concentrations during diabetic pregnancy may be due to its secretion by adipocytes in presence of elevated estrogen [72] and by placenta [73]. In fact, leptin, acting as a signal for sufficient energy supply, is persistently increased in women with GDM after delivery and associated with hyperglycemia and insulin resistance [69]. Hence, leptin, as a pro-inflammatory factor, may contribute to the inflammatory state during gestational diabetes. In contrast, low leptin level in macrosomic babies may contribute to the weight gain, since leptin-deficient rodents [68] and human [74] have been shown to develop obesity.

### 6.2. Animal Models

In order to investigate the relationship between insulin resistance and inflammation along with the obesity-related parameters such as adiponectin and leptin and pro-inflammatory markers, we have very recently undertaken a study in insulin-resistant offspring born to streptozotocin-induced diabetic pregnant mice [75]. Adiponectin and leptin expression is positively correlated with the epididymal adipose tissue mass which decreases in insulin-resistant offspring of diabetic mice [75]. Hence, reduced adiponectin contributes to insulin resistance as this adipokine, an anti-inflammatory agent, has been shown to enhance insulin sensitivity [63, 76]. Insulin resistance induces high expression of IL-6 and TNF-*α* mRNA in epididymal adipose tissue [75]. Adipose tissue secrete IL-6 and TNF-*α* during insulin resistance [77], and high levels of TNF-*α* and IL-6 may downregulate the expression of adiponectin [64] (Figure 1). During insulin resistance, increased IL-6 might not only diminish insulin sensitivity by suppressing insulin signal transduction but also interfere with anti-inflammatory effect of insulin, and might favour inflammation during insulin resistance [57]. 

All these clinical and experimental observations suggest that TNF-*α* and IL-6 may be involved in the pathogenesis of insulin resistance, and there is a positive correlation between insulin resistance and inflammation in GDM and macrosomia.

## 7. Immune System Modulation during Maternal Diabetes and Macrosomia

There is a growing body of evidence that suggests the implication of a pathological role of immune system and inflammation in type 1 diabetes, type 2 diabetes, and GDM. Indeed, T-cell-derived cytokines are involved in the autoimmune destruction of pancreatic islet cells leading to type 1 diabetes [22] whereas type 2 diabetes is associated with a generalized activation of innate immune system, in which there is a chronic, cytokine-mediated state of low-grade inflammation [78–80]. Moreover, evidence from human and experimental models suggests that a shift between Th1 and Th2 cells may modulate the severity of type 1 diabetes [22, 41], in which Th1 cytokines are highly produced during the islet inflammatory response and may partially explain the ability of CD4^+^ T cells to cause *β*-cell destruction [23]. In the nonobese diabetic (NOD) mouse, the most common animal model of human type 1 diabetes, it is observed an autoimmune destruction of pancreatic *β* cells, mediated by both CD4^+^ and CD8^+^ T cells [23]. 

**Figure 2:**
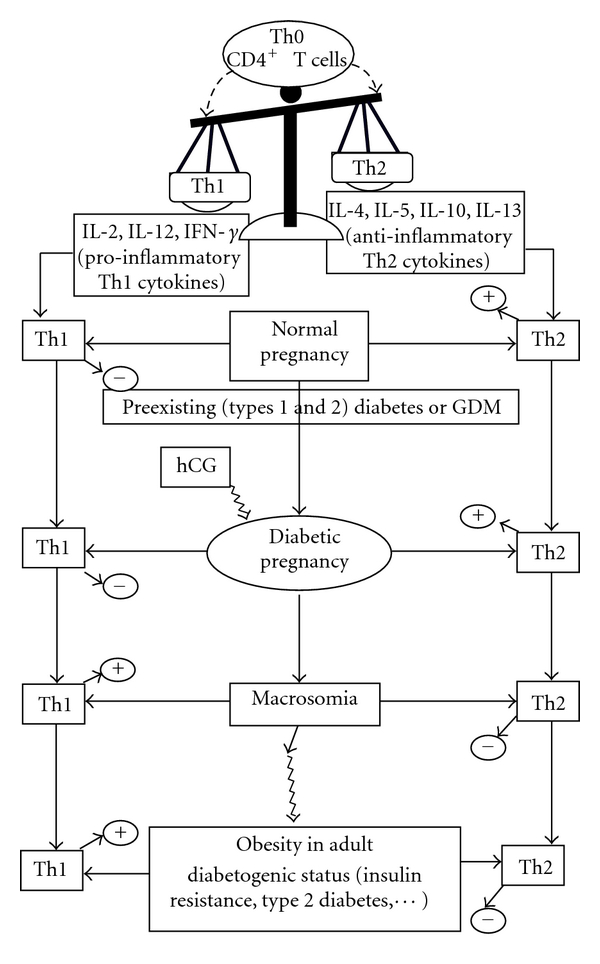
Naïve CD4^+^ T-helper (Th0) cells can be differentiated into either Th1 cells, producing pro-inflammatory cytokines (IL-2, IL-12, IFN-*γ*) or Th2 cells, secreting anti-inflammatory cytokines (IL-4, IL-10, IL-5, IL-13). In normal pregnancy as well as in diabetic pregnancy, the Th1/Th2 balance shifts towards a protective Th2 phenotype, whereas it shifts towards a pro-inflammatory Th1 phenotype in macrosomia as well as in obesity. Moreover, reproductive hormone like hCG may contribute to the low Th1 phenotype in diabetic pregnancy associated with the successful pregnancy. Th, T helper cells; hCG, human chorionic gonadotrophin; GDM, gestational diabetes mellitus; (+) upregulation; (−) downregulation.

Besides, in normal pregnancy, Th1 cytokines are downregulated, whereas Th2 cytokines are upregulated [81, 82], in animals as well as in humans. Even though the induction of type 1 diabetes is closely associated with high expression of Th1 cytokines, IFN-*γ* in particular [81], experimental and clinical studies reveal that, in pregnancy complicated with type 1 diabetes, Th1 cytokines are downregulated in diabetic pregnant rats and in women with GDM [16, 29]. Type 1 diabetic pregnancy in rats and GDM in women also induce increased level of IL-10, a Th2 cytokine [16, 29]. The level of IL-4 (another Th2 cytokine) is either decreased in diabetic animals [16, 83] or unchanged in GDM patients [16], due to the presence of diabetes [25]. Diminished Th1 cytokines and increased IL-10 (a Th2 cytokine) may be implicated in maintaining the pregnancy in diabetic rats and GDM patients (Figure 2). In fact, the shift of Th1/Th2 ratio to a protective Th2 phenotype during pregnancy (in animal and humans) has been shown to encourage vigorous production of antibodies which not only combat infections during pregnancy but also offer passive immunity to fetus [84]. On the other hand, the downregulated Th1 profile in diabetic pregnant animals and GDM patients (associated with successful pregnancy) may be contributed by elevated levels of reproductive hormones like hCG (human chorionic gonadotrophin) whose administration is known to diminish the production of Th1 cytokines [85].

As far as macrosomia is concerned, evidence in animals and humans reveals that macrosomia and obesity are associated with the shift of Th1/Th2 ratio to the Th1 phenotype [16, 29].

To sum up, it is interesting to note that diabetes during pregnancy in animals and human shifts the balance of Th1/Th2 cells to a protective Th2 phenotype, whereas, in macrosomic and obese offspring of diabetic dams, the Th1/Th2 balance is shifted to a pro-inflammatory Th1 phenotype (Figure 2). This upregulated Th1 profile in obese offspring may confer to these animals a potential pro-inflammatory and “diabetogenic status,” as revealed by the hyperglycemia and hyperinsulinemia observed in these animals in adulthood [27]. All these observations presume the long-term effects of maternal diabetes on the health of the offspring during their adulthood.

## 8. Problems Associated with Obesity and Diabetes during Pregnancy

The prevalence of obesity is increasing across the world [86]. Using the WHO criteria, obesity can now be defined by three grades of severity: grade I obesity with 30 ≤ BMI ≤ 34.9 kg/m^2^, grade II or severe obesity with 35 ≤ BMI ≤ 39.9 kg/m^2^, and grade III or massive obesity with BMI ≥ 40 kg/m^2^. Overweight is defined as 25 ≤ BMI ≤ 29.9 kg/m^2^. People whose BMI is comprised between 18.5 and 24.9 kg/m^2^ are considered as being normal weight (subjects with a BMI below 18.5 kg/m^2^ are considered as being underweight). 

Obesity pandemic is affecting all groups of age, including children, adolescents, young adults, and adults [87, 88]. Consequently, there are a growing number of obese women who are becoming pregnant. 

To an extent, obesity epidemic is explained by the increase in availability and consumption of energy-dense foods and a reduction in physical activity. However, there are additional putative factors which may explain the entire explosion in obesity prevalence [89]. These putative contributors operate through genetic factors, reproductive behaviors, and/or the intrauterine milieu, matters of importance for those involved with obesity and diabetes in pregnancy [89]. 

 Naturally in normal pregnancy, there is a physiological trend of insulin resistance from the second trimester. But in context of obesity, hyperinsulinemia associated with insulin resistance leads to the occurrence of GDM [90]. Moreover, it is known that obesity is linked to high adiposity and hyperlipidemia [57]; however, central fat, rather than peripheral adiposity, is more associated with insulin resistance, a predisposing factor to GDM [91, 92]. 

On the other hand, some investigators have recently found that maternal weight gain during pregnancy increases the offspring birth weight and the offspring's risk of obesity later in life, independently of genetic factors [93]. Similarly, Roman et al. [94] have found that maternal obesity was significantly associated with complications on the mother as well as on her baby: maternal obesity leads to the need for oral hypoglycemic agents or insulin, development of pregnancy-related hypertension, interventional delivery, and cesarean delivery. Adverse neonatal outcomes were also significantly increased including stillbirth, macrosomia, shoulder dystocia, hypoglycemia, and jaundice [94]. However, recent investigations report that macrosomia appears to be the predominant adverse outcome in cases of GDM [15]. Maternal obesity is an additional risk factor for complications, regardless of diabetes status [15].

To sum up, there are differences between women with GDM and obese women in pregnancy, as women with GDM possibly have greater macrosomia (even after treatment). There is clearly far greater neonatal hypoglycemia and jaundice among the offspring of women with GDM than those from obese women, but this observation and other data have to be interpreted with caution as some women may have had undiagnosed preexisting diabetes [5].

## 9. Management of Long-Term Impact of Maternal Obesity and Diabetes on the Offspring

Only few studies are available on pregnancy intervention for maternal diabetes and obesity during pregnancy. Weight loss may not be recommended during pregnancy [95]. However, evidence suggests that obesity's surgery-associated weight loss may be linked to less obesity in the offspring [96]. The outcomes of pregnancy complicated with maternal obesity or preexisting diabetes (mainly type 1 and type 2 diabetes, including undiagnosed type 2 diabetes) and GDM depend upon the intensity of treatment. Some studies show that treatment of GDM appears to reduce the risk of postpartum depression symptoms in the mothers [97]. Untreated GDM may be associated with a 2-fold risk of increased weight in offspring at 5–7 years [98]. Some observations show that more intensive treatment with insulin in GDM might be associated with less adiposity in offspring by 2 years 8 months [99]. However, randomized clinical studies of the management of GDM showed that treatment of GDM reduced macrosomia at birth, but did not show a reduction in BMI at the age 4-5 years [15, 100]. 

Nutritional strategies have also been proposed, since experimental and clinical evidence prove the beneficial effects of omega-3 fatty acid consumption during diabetes [101, 102]. Epidemiological studies have shown low incidence of inflammatory diseases in Greenland Eskimos and Japanese people [103], and this is attributed to large consumption of cold water marine fish that contain omega-3 fatty acids [104, 105]. In experimental studies, omega-3 fatty-acid enriched diet improves the hyperlipidemia induced by diabetic pregnancy and macrosomia [1, 27, 34]. Diabetic pregnancy and macrosomia are associated with increased oxidative stress (see above), and omega-3 fatty acid consumption also restores the decreased antioxidant status of diabetic pregnant animals and their macrosomic and obese offspring [1]. Moreover, omega-3 fatty acid enriched-diet exerts beneficial effects on immune system by promoting a protective Th2 phenotype during diabetic pregnancy and macrosomia [29]. Consumption of omega-3 fatty acid also prevents long-term metabolic abnormalities associated with macrosomia [27, 34]. 

Furthermore, many studies have reported that dietary supplements by vitamins and minerals prevent or, at least, attenuate organic deterioration caused by an excessive oxidative stress in diabetic subjects [106, 107]. As regards recent observations that many obese women are serologically vitamin D deficient, it is now recommended in the UK that Vitamin D supplementation may be provided for all women with a prepregnancy BMI of 30 kg/m^2^ [108].

## 10. Conclusion

Maternal diabetes or obesity during pregnancy appears to be an important risk factor for fetal obesity or macrosomia. Alterations in macrosomic infants persist postnatally and conduct to several abnormalities including the development of insulin resistance, obesity, diabetes, and metabolic syndrome at adulthood. Management of GDM and maternal obesity, including nutritional strategies, may have real improvement on maternal health and offspring in the future life.

## Abbreviations

GDM: Gestational diabetes mellitus
BMI: Body mass index
CD: Cluster of differentiation
HAPO: Hyperglycemia and adverse pregnancy outcomes
hCG: human chorionic gonadotrophin
IL: Interleukin
IFN: Interferon
AA: Arachidonic acid
LA: Linoleic acid
NOD: Nonobese diabetic
OGTT: Oral glucose tolerance test
ORAC: Oxygen radical absorbance capacity
TNF: Tumor necrosis factor
Th cells: T helper cells
TBARS: Thiobarbituric acid-reactive substances
TG: Triglyceride
TC: Total cholesterol
SOD: Superoxide dismutase.
